# Food Bolus Masquerading as a Cardiac Mass on Echocardiogram

**DOI:** 10.7759/cureus.17872

**Published:** 2021-09-10

**Authors:** Abhilash Makkar, Talhah Siraj, Stacy Zimmerman, David Evans, Eric Landa, Ismail Ganim, Suporn Sukpraprut-Braaten, Stephen D Wagner, Aaliya Abhilash

**Affiliations:** 1 Internal Medicine, Unity Health - White County Medical Center, Searcy, USA; 2 Cardiology, Unity Health - White County Medical Center, Searcy, USA; 3 Internal Medicine, Unity Health, Searcy, USA; 4 Statistics, Unity Health - White County Medical Center, Searcy, USA; 5 Senior, Cabot High School, Cabot, USA

**Keywords:** hiatal hernias, transthoracic echocardiography, transesophageal echo, intracardiac mass, myxomas

## Abstract

An echocardiogram is the most utilized imaging modality in the evaluation of patients with intracardiac masses. Of these, left atrial myxomas are the most common intracardiac tumors in which diagnostic recognition is important for appropriate management. This paper demonstrates a case study on how a proper physical exam along with proper imaging modalities may result in avoiding misdiagnosing patients with large sliding hiatal hernias who present with the complaint of chest pain.

This case reveals a food bolus within a sliding hiatal hernia masquerading as an intracardiac mass. Cardiac masses can potentially be misdiagnosed using transthoracic echocardiography (TTE). While transthoracic echocardiography is uniquely resourceful as a point-of-care modality, it can be challenging to confirm whether an identified mass is attached to the myocardium or other cardiac or intrathoracic structures. The patient exhibited symptoms compatible with a cardiac mass. Primarily, the physical examination is vital in determining whether the patient should be referred for additional diagnostic modalities to assess for a cardiac mass. Auscultation of bowel sounds on the cardiac examination is consistent with a suspected hiatal hernia.

## Introduction

Transthoracic echocardiography (TTE) is the most commonly utilized imaging modality in the evaluation of patients with intracardiac masses, such as left atrial myxomas. Such tumors necessitate diagnostic recognition for appropriate management and treatment. On the other hand, diaphragmatic hernias can be hiatal or Morgagni and can appear like cardiac masses [1]. Hiatal hernias may demonstrate diagnostic challenges as many may be incorrectly diagnosed as cardiac masses using TTEs [1]. This case demonstrates how proper physical exam and imaging modalities may avoid misdiagnosing patients with large sliding hiatal hernias who present with the complaint of chest pain.

## Case presentation

**Figure 1:**
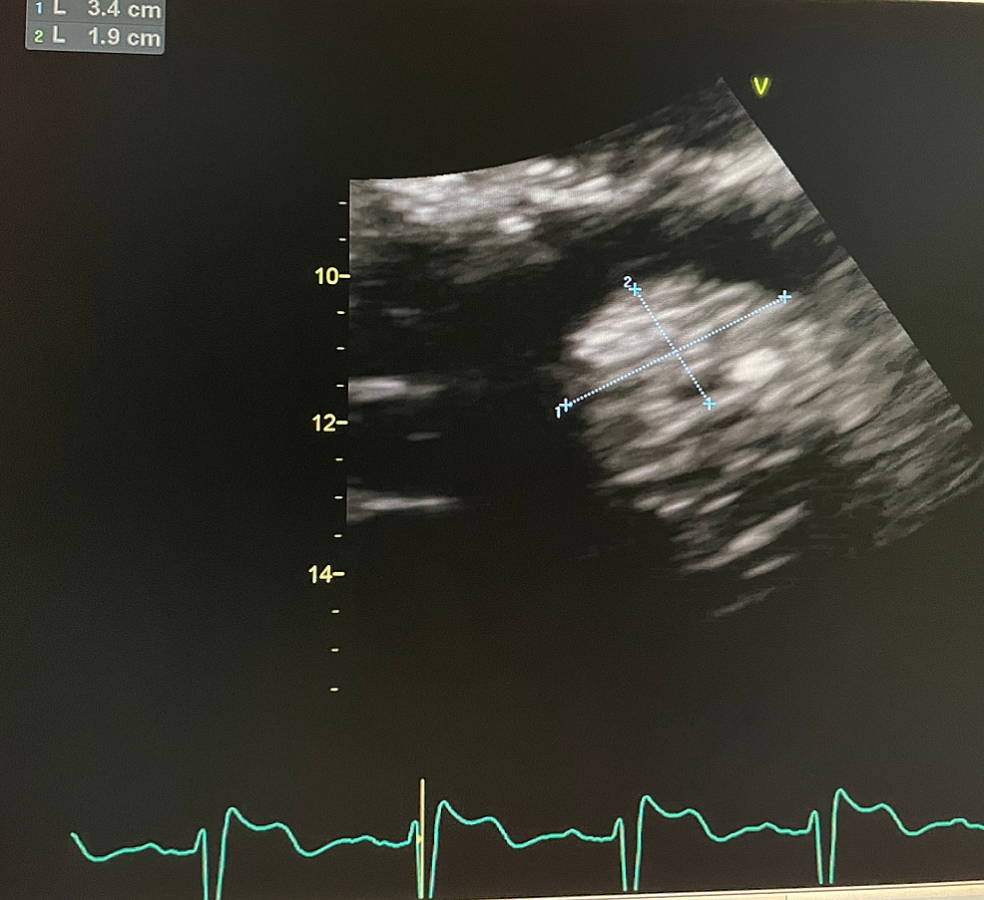
Suspected myxoma revealed by TTE TTE: Transthoracic echocardiogram

An 89-year-old female with a past medical history of hypertension and hyperlipidemia presented to the emergency department complaining of chest pain. The patient stated that the chest pain had begun soon after waking up. It was located in the substernal region of the chest with radiation to her left arm and was an 8/10 constant pain. The pain was not associated with any nausea, vomiting, or other symptoms. Two murmurs were auscultated: a systolic ejection murmur grade 2/6 and a late peaking musical systolic murmur grade 3/6 both heard along the left upper sternal border on the physical exam, along with bowel sounds in the chest. A stat chest X-ray and EKG were done which resulted as unremarkable. A transthoracic echocardiogram (TTE) was scheduled for further evaluation. Initial troponin was less than 0.01 ng/mL. A TTE (Video 1) revealed what appeared to be an intracardiac mass that was suspected to be an atrial myxoma (Figure 1).

**Video 1:** Suspected myxoma on TTE TTE: Transthoracic echocardiogram

The patient was scheduled for a transesophageal echocardiogram (TEE) the following morning (Video 2).

**Video 2:** TEE on the following morning did not demonstrate a cardiac mass TEE: Transesophageal echocardiogram

**Figure 2:**
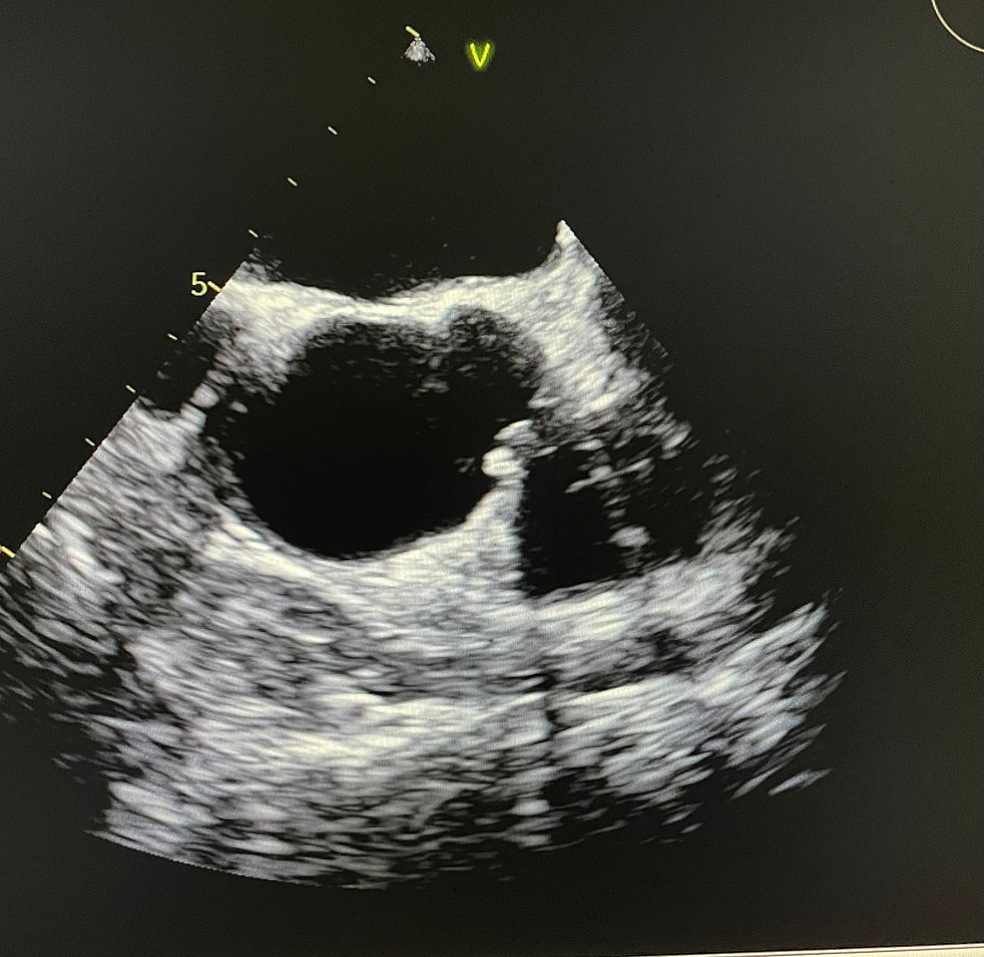
TEE on the following morning did not demonstrate a cardiac mass TEE: Transesophageal echocardiography

The TEE did not demonstrate a cardiac mass. Upon further investigation, the TEE determined that the patient had a large sliding hiatal hernia without a food bolus. Since the patient was NPO (*nil per os*) overnight, it was concluded that the hiatal hernia contained a food bolus when the TTE was performed, thus masquerading as an intracardiac mass (Figure 2).

## Discussion

Cardiac masses are susceptible to misdiagnosis using a TTE. While TTE is uniquely resourceful as a point of care modality, it can be challenging to confirm whether an identified mass is attached to the myocardium or other cardiac or intrathoracic structures [2]. The patient exhibited symptoms and physical exam findings compatible with a cardiac group. A TTE was a natural next step in evaluation. With the identification of what appeared to be a cardiac mass, further evaluation was necessary, hence a TEE was ordered. There were some features in the case that makes a cardiac mass low on the differential. Reports have also shown that based on differential diagnoses that may not include a TTE, a hiatal hernia can mimic a pericardial lipoma [2]. Such cases are, however rare, which makes overlooking them easy. Typically, if a cardiac mass is the culprit, we would auscultate a diastolic murmur with a plop. Additionally, in conjunction with the bowel sounds heard in the chest, if a chest X-ray was ordered, we would be able to identify the large hernia pushing against the heart. 

Cardiac masses are rare in elderly female patients [3]. Primarily, the physical examination is vital in determining whether the patient needs additional diagnostic modalities to assess for a cardiac mass. Auscultation of bowel sounds on the cardiac exam is consistent with a suspected hiatal hernia [2]. Diagnostic modalities like echocardiography and CT scans aid in broadening the differential diagnosis to determine whether a suspected mass is, in fact, of cardiac origin [4]. Before referring patients for an advanced imaging evaluation of a suspected cardiac group, a physical examination with primary imaging modalities, particularly chest X-rays, should be conducted and reviewed.

Hernias are classified differently depending on the presented symptoms, physical examination, and imaging. A hiatal hernia details the displacement of abdominal contents into the thoracic cavity through the diaphragmatic opening [5]. Symptoms of hernias vary depending on the type, but chest pains are relatively mild symptoms [5]. The patient’s signs and revelations from examinations were consistent with herniation. She had systolic dysfunction, a well above average chest pain, bowel sounds in her chest, and an unremarkable EKG. The TTE conducted on the patient revealed an intracardiac-like mass suspected to be an atrial myxoma. Although hernias are highly prevalent, echocardiography reveals very few [6]. As such, further imaging modalities would have been necessary such as a TEE. 

Hiatal hernias commonly present as acute chest pain, yet echocardiograms can give them as cardiac entities. Physical examination and agile testing are necessary for correct diagnosis when echocardiograms show left atrial mass structures [6]. Hernias of various classifications require specified testing because some tests may be inadequate, especially when patients have a significant medical history. For instance, high-resolution manometry (HRM), which employs pressure gradients, is more sensitive and specific in diagnosing hernias than barium swallow and endoscopy [5]. Whenever physical examinations do not suffice, combining different techniques is vital.

TTE is a preferred non-invasive investigation approach to cardiac pathologies. Case studies and reports have consistently highlighted that using echocardiograms for cardiac anatomy-related issues, gastrointestinal pathology mimicking cardiac conditions and entities have become a characteristic feature [1]. Hiatal hernia is generally asymptomatic but when severe can manifest as chest pain and syncope, which are also common symptoms of cardiac masses on TTEs. Techniques such as cardiac magnetic resonance imaging (MRI) have better spatial resolution and efficacy in describing atrial masses [1]. In this case study, a food bolus masqueraded as a cardiac mass and it was absent after a night without food and drink intake. Consequently, caution is necessary for accurate diagnosis.

## Conclusions

Hiatal hernias manifest as left atrial masses when evaluated using the transthoracic echocardiography diagnostic technique. Consequently, numerous misdiagnosis cases have occurred in patients due to the similarities in the manifestation of hiatal hernias and cardiac masses. To avoid the diagnostic error that can jeopardize patients’ trust in healthcare or attract legal investigations, physical examinations combined with imaging modalities can improve diagnosis. Additionally, enhanced imaging such as MRIs, CTs, chest radiographs, and endoscopy and barium swallow help eliminate other sources and confirm hiatal hernia. Despite being a common evaluation technique, an echocardiogram only does not suffice in the diagnosis of hiatal hernias.
